# The Causal Nexus Between Different Feed Networks and Defected Ground Structures in Multi-Port MIMO Antennas

**DOI:** 10.3390/s24227278

**Published:** 2024-11-14

**Authors:** Merve Tascioglu Yalcinkaya, Shahanawaz Kamal, Padmanava Sen, Gerhard P. Fettweis

**Affiliations:** Barkhausen Institut, 01067 Dresden, Germany; shahanawaz.kamal@barkhauseninstitut.org (S.K.); padmanava.sen@barkhauseninstitut.org (P.S.); gerhard.fettweiss@barkhauseninstitut.org (G.P.F.)

**Keywords:** antenna, defected ground structure (DGS), feed network, multiple input multiple output (MIMO), slotted complementary split ring resonator (SCSRR), envelope correlation coefficient (ECC), diversity gain (DG), channel capacity loss (CCL), mean effective gain (MEG)

## Abstract

Multiple input multiple output (MIMO) antennas have recently received attention for improving wireless communication data rates in rich scattering environments. Despite this, the challenge of isolation persists prominently in compact MIMO-based electronics. Various techniques have recently emerged to address the isolation issues, among which the defected ground structure (DGS) stands out as a cost-effective solution. Additionally, selecting the appropriate feed mechanism is crucial for enhancing the key performance indicators of MIMO antennas. However, there has been minimal focus on how different feed methods impact the operation of MIMO antennas integrated with DGS. This paper begins with a comprehensive review of diverse antenna design, feeding strategies, and DGS architectures. Subsequently, the causal relationships between various feed networks and DGSs has been established through modeling, simulation, fabrication, and measurement of MIMO antennas operating within the sub-6 GHz spectrum. Particularly, dual elements of MIMO antennas grounded by a slotted complementary split ring resonator (SCSRR)-based DGS were excited using four standard feed methods: coaxial probe, microstrip line, proximity coupled, and aperture coupled feed. The influence of each feed network on the performance of MIMO antennas integrated with SCSRR-based DGSs has been thoroughly investigated and compared, leading to guidelines for feed network selection. The coaxial probe feed network provided improved isolation performance, ranging from 16.5 dB to 46 dB in experiments.The aperture and proximity-coupled feed network provided improvements in bandwidth of 38.7% and 15.6%, respectively. Furthermore, reasonable values for envelope correlation coefficient (ECC), diversity gain (DG), channel capacity loss (CCL), and mean effective gain (MEG) have been ascertained.

## 1. Introduction

In the modern era, wireless communication has become an indispensable part of human life, which has paved the way for the development of advanced communication systems [1,2,3,4]. Particularly, compact antennas with high gain and wide bandwidth (BW) are essential for rendering reliable communication links [5,6,7,8]. MIMO antennas have attracted significant attention in research because they satisfy the aforementioned requirements in addition to providing higher data rates and extended throughput capabilities [9,10,11,12,13,14,15,16,17]. However, MIMO antennas are associated with the challenge of isolation, which typically arises in compact devices where multiple radiating elements are placed in close proximity [18,19,20,21,22,23,24]. Descriptively, isolation represents one of the main bottlenecks of MIMO antennas because it leads to undesirable effects, including reduced antenna radiation efficiency (RE), impedance mismatch, and poor diversity performance.

Several studies reviewing MIMO antenna isolation enhancement strategies have been published in recent years [25,26,27,28,29,30,31,32,33,34,35]. In general, cost-effective techniques for improving MIMO antenna isolation include neutralization lines [36,37,38,39], decoupling networks [40,41,42,43] and parasitic structures [44,45,46,47]. Their operating mechanisms involve the introduction of an extra coupling current that ultimately cancels out the original coupling current between the antenna elements. However, these solutions degrade some of the key performance metrics of MIMO antennas. Composite media created to exhibit distinctive electromagnetic properties, such as metamaterials [48,49,50,51,52,53] and EBG structures [54,55,56,57], are noteworthy solutions that can achieve high isolation while maintaining the desired overall performance of MIMO antennas. Nonetheless, these alternatives necessitate complex layouts and high fabrication costs. On the contrary, the DGS represents a cost-effective and easy-to-fabricate solution for a wide range of applications [58,59,60,61].

It has previously been demonstrated that determining a feeding mechanism for microstrip antennas is a crucial choice because it has an immediate impact on the device’s overall performance [62,63,64]. The four most utilized feed procedures include coaxial probe [65,66,67], microstrip line [68,69,70], proximity-coupled [71,72,73], and aperture-coupled [74,75,76]. However, the impact of different feed networks on the performance of MIMO antennas integrated with DGSs has never been considered. Consequently, this research aimed to determine the causal relationships between different feed networks and DGS-based MIMO antennas. The sub-6 GHz spectrum of 5G networks, currently at the cutting edge of wireless technology, was selected as the desired operating frequency band for this study [77,78,79]. Descriptively, 5.8 GHz was the intended $f_{c}$ for both radiating and DGS elements. Rectangular patches were taken into consideration as the MIMO antenna’s radiating elements owing to their lowered design complexity, and noteworthy antenna key performance indicators that have been determined in the past by many researchers [63,80]. In addition, the SCSRR was chosen as the DGS configuration in recognition of their noteworthy advantages, which are covered in further detail in the next section [81,82,83]. Note that the sole objective of these MIMO antenna specifications and their operational spectrum is a starting point to begin the detailed investigations. Overall, this study is significant, since it provides a clear road map to determine the antenna performance enhancement capabilities, design complexity, cost analysis, and feasibility for integration with diverse technologies.

The major contributions of this paper are summarized below:

1. A thorough review with succinct summary on the antenna feeding techniques and DGS layouts.
2. Modeling of the dual element rectangular patch antenna and SCSRR-based DGS for operation in the sub-6 GHz spectrum.
3. The four (one at a time) feed procedures, namely, coaxial probe, microstrip line, proximity-coupled, and aperture-coupled, were applied to a dual port antenna integrated with an SCSRR-based DGS for the simulation studies of antenna as well as diversity parameters.
4. Validation of simulation studies by fabrication and measurements of four dual-port antennas integrated with an SCSRR-based DGS and fed via four distinct feed methods.
5. Comparison of different feed networks of SCSRR-based DGS MIMO antennas in terms of BW, peak isolation, and peak gain.

This paper is outlined as follows. Section 2 covers the fundamental background of the antenna design methodology, feeding techniques, and DGS layouts. Section 3 presents the simulation results of SCSRR-based DGS MIMO antennas with different feed networks, and it analyzes their performance metrics (i.e., ECC, CCL, and MEG). Section 4 describes the measured results of the fabricated antennas. Section 5 benchmarks this work with existing research publications. Section 5 benchmarks this work with existing research publications. Moreover, it illustrates the comparison of different feed networks of CSRR-based DGS MIMO antennas in terms of BW, peak isolation, and gain. Finally, Section 6 provides the conclusions and future research directions.

## 2. Fundamental Background

### 2.1. Antenna Design Methodology

The dimensions of a single element of the MIMO antenna can be estimated from the following equations [62]:

(1) $$W=\frac{c_{0}}{2f_{r}}\sqrt{\frac{2}{\varepsilon_{r+1}}}\;$$

(2) $$\varepsilon_{reff}=\frac{\varepsilon_{r}+1}{2}+\frac{\varepsilon_{r}+1}{2}{\left[1+12\frac{h}{W}\right]}^{-1/2},\;W/h>1$$

where, *W* is the width of patch, *h* is the height of the dielectric substrate, $\varepsilon_{reff}$ is the effective dielectric constant of the substrate, and $\varepsilon_{r}$ is the dielectric constant of substrate.

The dimension of the patch may be extended on each end by ΔL due to fringing fields, which is the function of the effective dielectric constant $\varepsilon_{reff}$ and width-to-height ratio W/h>1.

(3) $$\frac{\Delta L}{h}=0.412\frac{(\left(\varepsilon_{reff}\right)+0.3)\left(\frac{W}{h}\right)+0.264}{(\left(\varepsilon_{reff}\right)+0.258)\left(\frac{W}{h}\right)+0.8}$$

The length of the patch antenna can be calculated while employing the subsequent calculations [62].

(4) $$L=\frac{c_{0}}{2f_{r}\sqrt{\varepsilon_{reff}}}-2\Delta L$$

The length and width of the ground plane is given by the successive formulas.

(5) $$L_{g}=6h+L$$

(6) $$W_{g}=6h+W$$

### 2.2. Antenna Feeding Techniques

**Figure 1:**
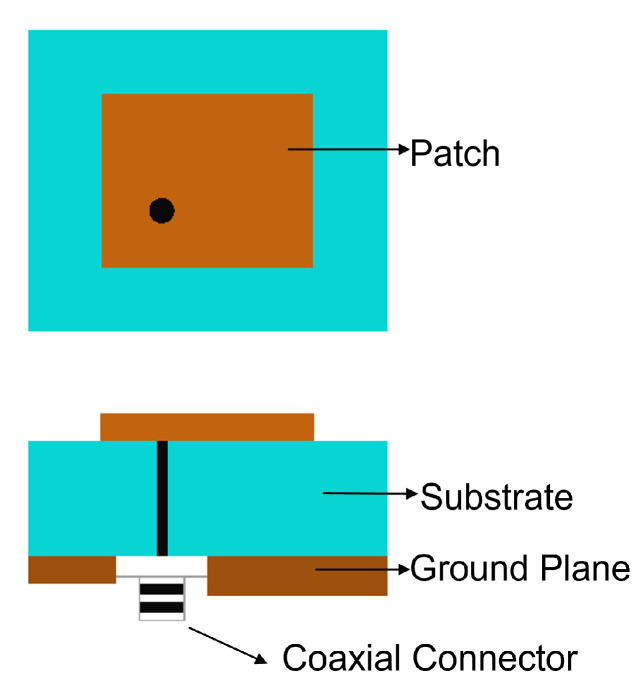
Coaxial probe feed.

**Figure 2:**
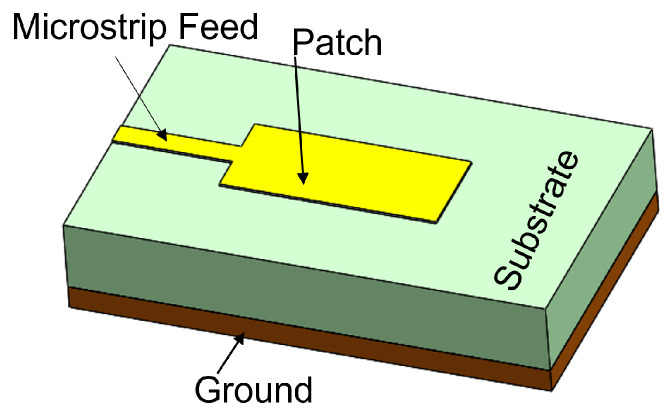
Microstrip line feed.

**Figure 3:**
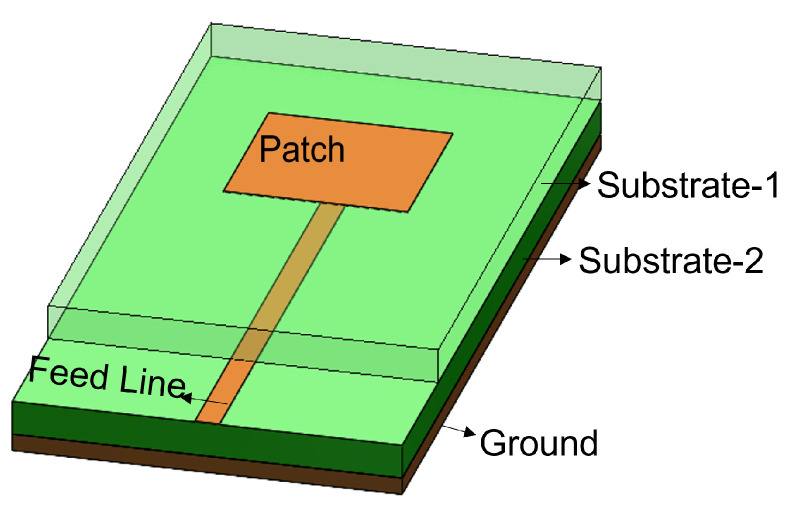
Proximity-coupled feed.

**Figure 4:**
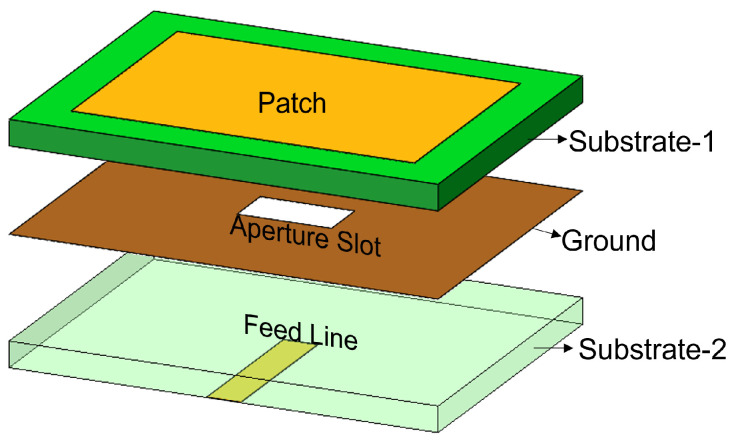
Aperture-coupled feed.

The feed technique represents an important design choice due to its strong impact on the antenna performance parameters such as BW, return loss, gain, etc. Feed techniques are categorized into two types as contacting feed and non-contacting feed techniques [62]. In the contacting feed technique, the power is fed directly to the radiating patch through the connecting line, whereas in the non-contact feeding technique, the power is transferred to the radiating patch through an electromagnetic coupling. In general, the microstrip line and coaxial probe feed are widely used as contact feeding techniques. The coaxial probe feed technique, which is popular in many applications of a microstrip patch antenna, consists of an inner conductor, an outer conductor, and a dielectric material in between. The inner conductor of the coaxial probe feed is connected to the patch, while the outer conductor is connected to the ground plane, as shown in Figure 1. In the microstrip line feed method, the conducting strip is connected to the microstrip patch, and its width is smaller than the patch’s width, as shown in Figure 2. On the other hand, the power is transferred between the radiating patch and the connecting element using the well-known non-contacting feed techniques of proximity coupling and aperture coupling, as demonstrated in Figure 3 and Figure 4. Each of the four feeding networks is explained in detail in [62]. Table 1 summarizes their advantages and disadvantages [62,84,85,86,87,88]. The comparison is primarily focused on the trade-off between fabrication complexity and antenna performance.

### 2.3. Defected Ground Structure Layouts

Historically, the DGS was designed as a unit dumbbell-shaped defect placed underneath a microstrip line to provide stop-band characteristics [89]. This configuration contributes to preventing electromagnetic waves from traveling down the microstrip line over a range of frequencies. Given that the mutual coupling effect mainly originates from surface waves that exist when MIMO elements share a common ground plane, the DGS can change the surface current distribution of the antennas to enhance isolation [23]. In other words, introducing the DGS in MIMO antennas results in the transformation of effective capacitance and inductance of the transmission line to operate as a filter [90]. Over the years, numerous DGS shapes have been obtained and reported in the literature for different applications [91,92,93,94]. These shapes have been deployed with the goal of suppressing the surface waves, attaining compact geometry, rejecting harmonics, and realizing the ease of fabrication. Table 2 provides a concise review of DGS techniques while highlighting their advantages and disadvantages [91,92,93,94]. Descriptively, DGS represents a low-cost method for realizing wave suppression and compact design in contrast to other techniques like EBG structures, circulators, and hybrid couplers.

**Figure 5:**
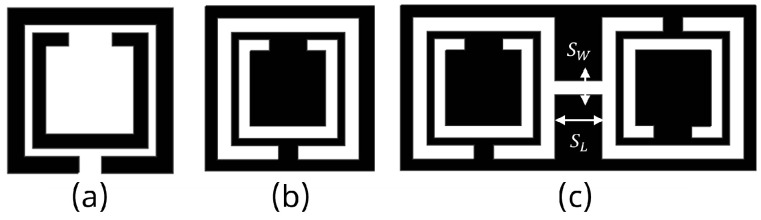
Metal (black) and substrate (white) regions of (**a**) SRR, (**b**) CSRR, and (**c**) SCSRR geometries.

**Figure 6:**
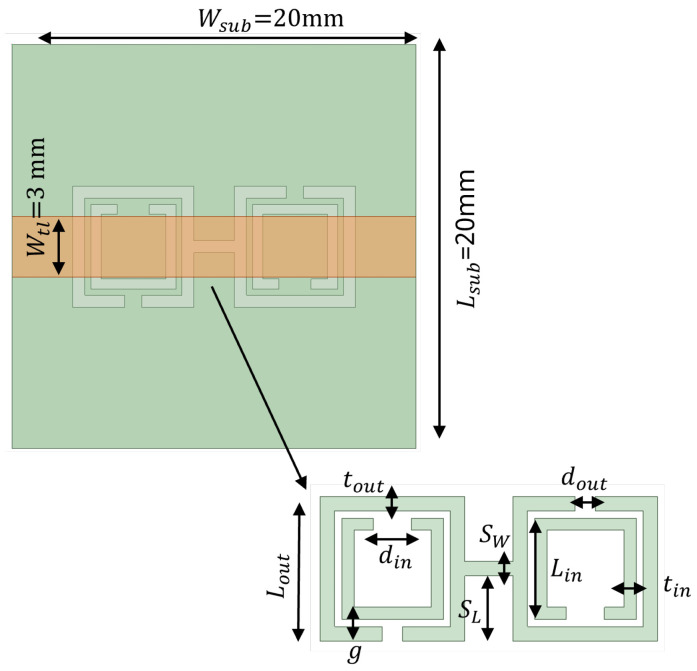
Configuration of SCSRR model and dimension of the SCSRR are provided in Table 3.

**Figure 7:**
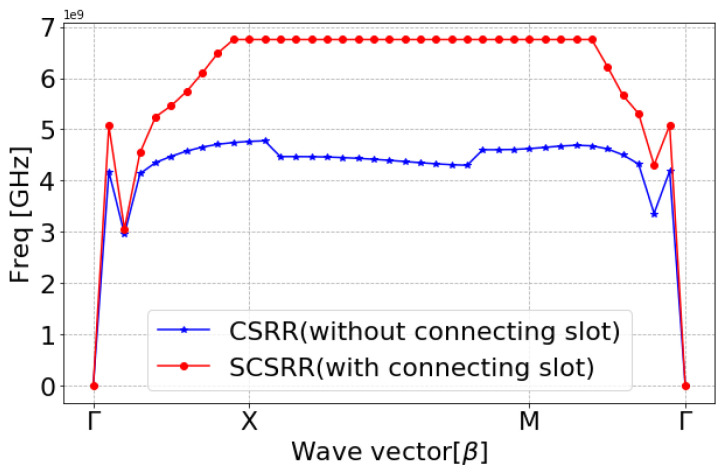
Dispersion diagram of CSRR and SCSRR (Freq = Frequency).

The SRR-based configurations have demonstrated substantial improvements in several antenna performance metrics within the wide spectrum of EBG/DGS designs [29,95,96]. Generally, SRR comprises of a pair of concentric metals with splits at the opposite opening direction on a substrate, as shown in Figure 5a. Their resonance characteristic stems from the regulated inductive–capacitive phenomenon, leading to an electric current flow through the metallic rings as well as gaps. The CSRR structure constitutes a negative variant of SRR, as illustrated in Figure 5b. Analogously, it operates like an inductive–capacitive resonating circuit, where the antenna’s resonance can be adjusted according to its dimensions. The SCSRR represents another form of SRR that is basically composed of two CSRR, as demonstrated in Figure 5c. The capacity of an SCSRR-based DGS to enhance out-of-band rejection without increasing the insertion loss stays remarkable [97,98]. Therefore, a SCSRR defect structure consisting of two rectangular CSRRs connected with a slot of $S_{W}$ and $S_{L}$, as shown in Figure 6, was considered for investigation. The SCSRR structure was designed to operate at 5.8 GHz, at the same desired operating frequency of the MIMO antenna. The filtering characteristics of the proposed SCSRR structure were studied by utilizing the eigenmode analysis feature of the ANSYS High-Frequency Structure Simulator (HFSS) software 2020 R2. The Brillouin diagram for an unit cell SCSRR structure is plotted along the wave vector β of the periodic structure in Figure 7. Strong rejection characteristics were obtained for the SCSRRs, where the bandgap zone (4.5–6.5 GHz) is clearly seen within the operating frequency range of 5–7 GHz.

**Figure 8:**
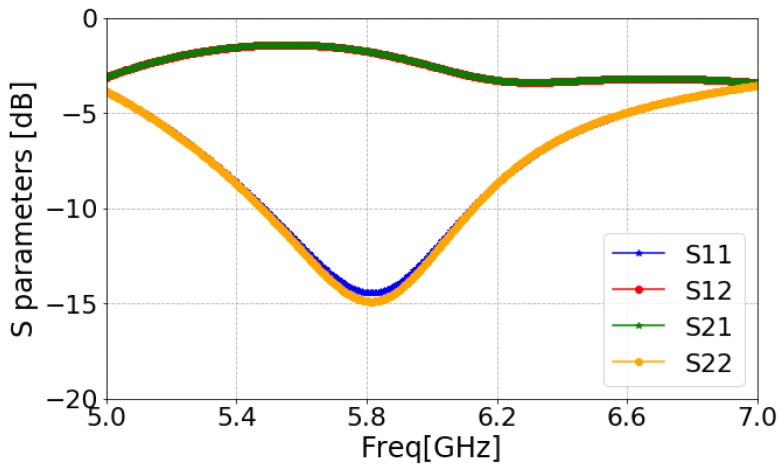
S-parameters of the SCSRR.

In order to provide the impact of the slot in comparison to the traditional CSRR unit cell, a comparative eigenmode analysis is shown in Figure 7. The bandpass behavior of the proposed unit cell of SCSRR (CSRR with slot) can be viewed as two cascaded filters. An alternative way to analyze the bandgap behavior of the SCSRR is obtaining the scattering (S) parameters of a microstrip transmission line placed over the substrate. The proposed MIMO antennas employ a Rogers RO4003C as the dielectric substratewith $\varepsilon_{r}$ of 3.55, tan*δ* of 0.0027, and thickness *h* of 1.52 mm. The SCSRR structure was etched on the ground plane below the microstrip line (see Figure 6), which has a width of 3 mm. The width of the microstrip line was designed to match the characteristic impedance of 50 Ω. Figure 8 shows the simulated S-parameters of the SCSRR. The simulation results prove that the band reject characteristics were accomplished at 5.8 GHz, which is challenging with conventional microstrip resonators.

## 3. Analysis of SCSRR-Based DGS MIMO Antennas with Different Feed Networks

In this section, the simulation results of MIMO antennas with different feeding networks are discussed. All of the proposed MIMO antennas were composed of two symmetrical and identical radiating elements working at 5.8 GHz. The total size of each MIMO antenna was 58 mm × 36 mm. The thickness *h* of the Rogers RO4003C substrate was chosen to be 1.52 mm. The center-to-center spacing between the two antennas was $d_{1}$ = 26 mm (0.5$\lambda_{0}$), where $\lambda_{0}$ is the free-space wavelength at 5.8 GHz. The proposed MIMO antenna designs were studied while considering full a ground plane, as well as SCSRR-based, DGS. The SCSRR design and dimensions described in Section 2 were considered for all cases. Accordingly, the antenna performance characteristics such as return loss, isolation, gain, BW, RE, and surface current distribution were investigated. Furthermore, the diversity parameters, including ECC, DG, MEG, and CCL have been verified to justify the MIMO antenna performance.

### 3.1. Coaxial Probe Feed

**Figure 9:**
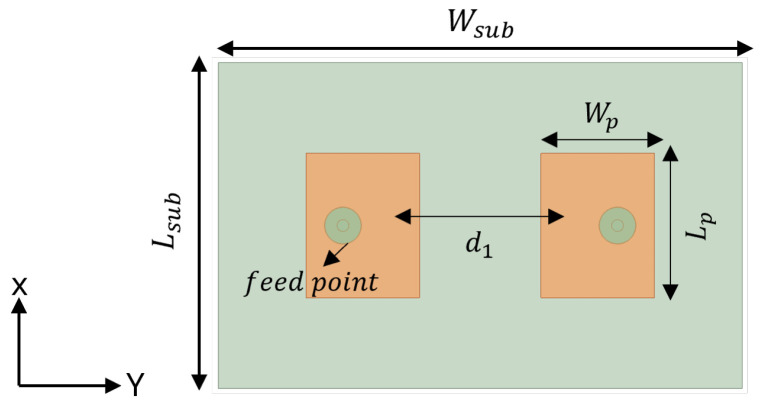
Coaxial probe feed MIMO antenna model without SCSRR-based DGS. Dimensions of antenna: L_*sub*_ = 36 mm, W_*sub*_ = 58 mm, L_*p*_ = 16 mm, W_*p*_ = 12.55 mm, d_1_ = 26 mm.

**Figure 10:**
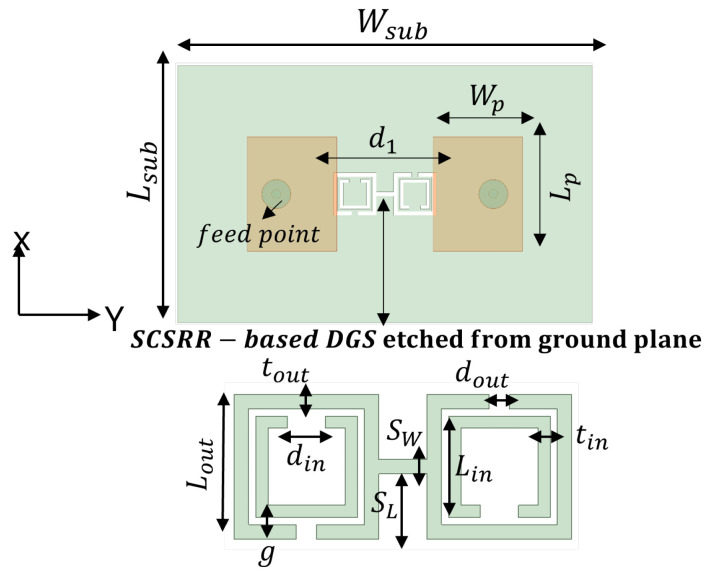
Coaxial probe feed MIMO antenna model with SCSRR-based DGS. Dimensions of antenna: L_*sub*_ = 36 mm, W_*sub*_ = 58 mm, L_*p*_ = 16 mm, W_*p*_ = 12.55 mm, d_1_ = 26 mm. Note: Dimensions of SCSRR are listed in Table 3.

To design a two-element MIMO antenna, the antenna design equations mentioned in Section 2 were used. The geometry of the proposed MIMO antennas with a coaxial probe feed is presented in Figure 9. The MIMO antenna was equipped with an SCSRR-based DGS (see dimensions Table 3), as illustrated in Figure 10. Their design and dimensions were determined from the equations detailed in Section 2.

**Figure 11:**
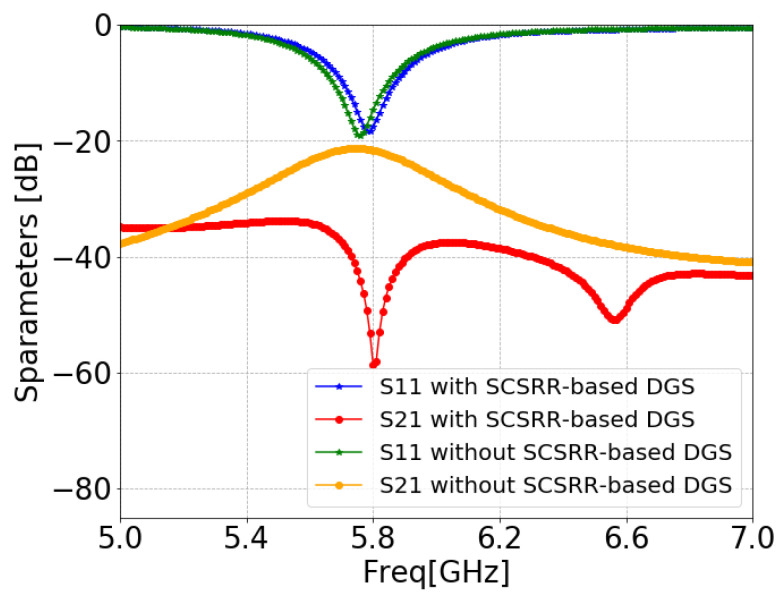
S-parameters of coaxial probe feed MIMO antennas with SCSRR-based DGS.

Figure 11 illustrates the ANSYS HFSS simulation results in terms of isolation ($S_{21}$/$S_{12}$) and return loss ($S_{11}$/$S_{22}$) of MIMO antenna with and without an SCSRR. The resonance frequency of the MIMO antenna without an SCSRR-based DGS had strong coupling, with an isolation value of 21 dB due to surface wave excitation. On the other hand, the MIMO antenna with anSCSRR-based DGS had suppressed surface waves, and simulations show that the peak isolation increased to 58 dB which is 31 dB higher than the without SCSRR-based DGS.

**Figure 12:**
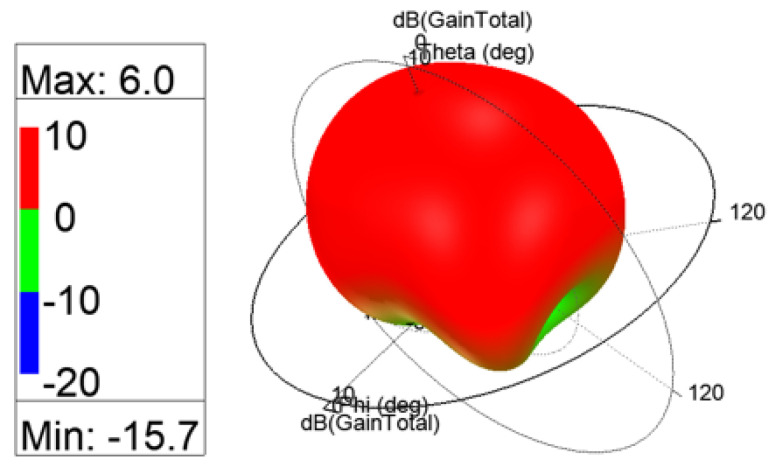
The 3D gain patterns of coaxial probe feed MIMO antennas with SCSRR-based DGS.

**Figure 13:**
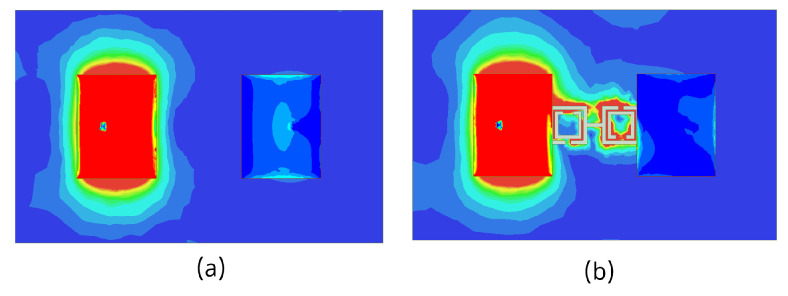
Current distribution of coaxial probe feed MIMO antennas with and without SCSRR-based DGS. (**a**) Without SCSRR-based DGS and (**b**) With SCSRR-based DGS.

The 3D gain patterns of the coaxial probe feed MIMO antennas with SCSRR-based DGS are illustrated in Figure 12. The distribution of surface currents on the ground plane with one antenna excited and the other terminated with a 50 Ω impedance is shown in Figure 13. According to Figure 13b, the suppression of the space wave by virtue of the bandgap filtering is clearly observed. In addition to this, the high concentration of the surface currents can observed in the loaded antenna, which resulted in only a slight shift in the resonance frequency of the patches, as depicted in Figure 11. The comparison of the antenna performance parameters with and without SCSRR-based DGS structures is provided in Table 4. The DGS-based antennas yielded relatively higher bandwidth in contrast to antennas without the DGS. The bandwidth enhancement effect was attributable to DGS structures’ property of decreasing the slope of the imaginary part of the input impedance of the antenna. However, the peak gain of antennas incorporating DGS was found to be lowered by up to 0.8 dBi, since the DGS structures increased the back lobe radiation. Yet again, the DGS-based antennas ascertained a gain of 6 dBi at 5.8 GHz. Furthermore, the peak isolation values of DGS-based antennas were improved from 16.5 dB to 58 dB over a bandwidth of 180 MHz.

### 3.2. Microstrip Line Feed

**Figure 14:**
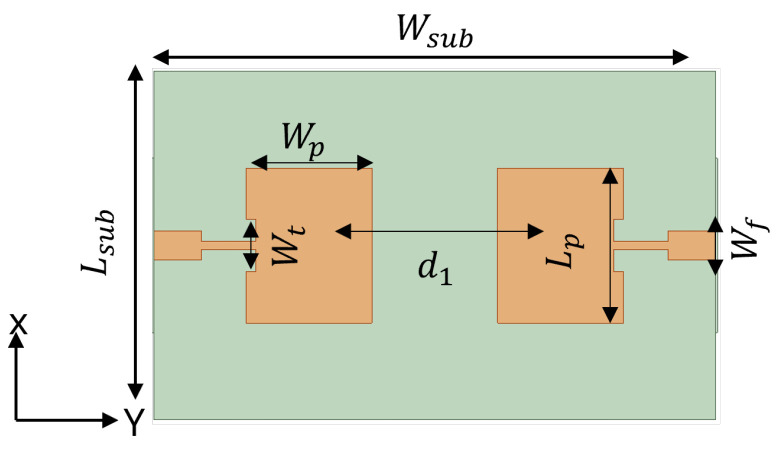
Microstrip line feed MIMO antenna model without SCSRR-based DGS. Dimensions of antenna: L_*sub*_ = 36 mm, W_*sub*_ = 58 mm, L_*p*_ = 16 mm, W_*p*_ = 13 mm, d_1_ = 26 mm, w_*f*_ = 3 mm, w_*t*_ = 0.8 mm.

**Figure 15:**
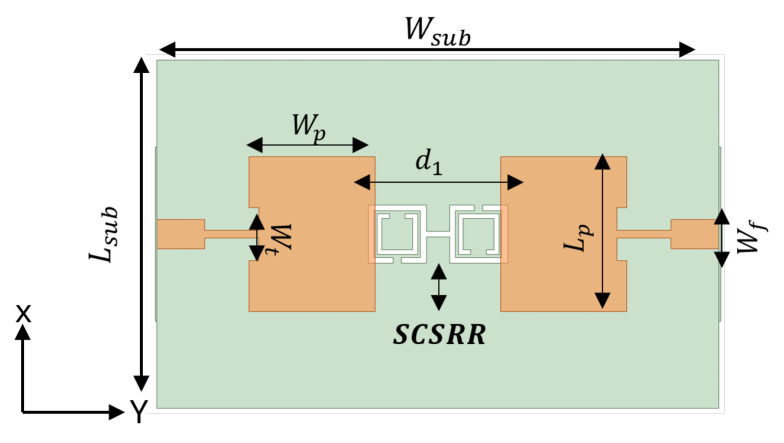
The microstrip line feed MIMO antenna model with SCSRR-based DGS. The dimensions of antenna are as follows: L_*sub*_ = 36 mm, W_*sub*_ = 58 mm, L_*p*_ = 16 mm, W_*p*_ = 13 mm, d_1_ = 26 mm, w_*f*_ = 3 mm, w_*t*_ = 0.8 mm. Note: Dimensions of SCSRR are listed in Table 3.

**Figure 16:**
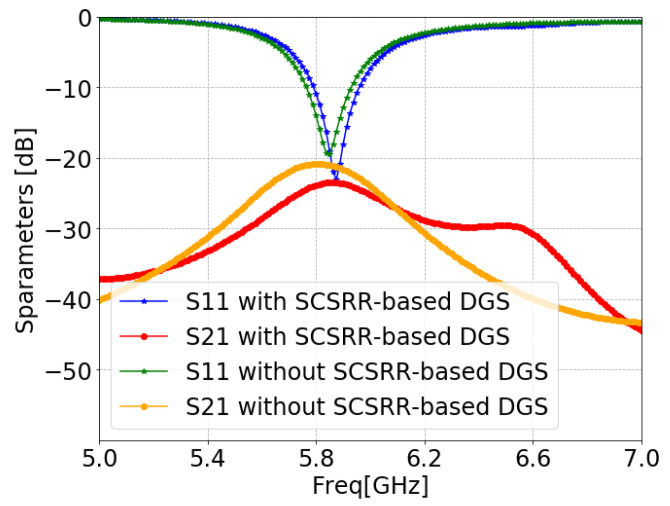
S-parameters of microstrip line feed MIMO antennas with and without SCSRR-based DGS.

The schematic view of the microstrip line feed MIMO antenna structure and its design parameters are illustrated in Figure 14. The SCSRR was implemented on the same MIMO antenna, as shown in Figure 15. The simulated S-parameters of microstrip line feed MIMO antennas with and without an SCSRR-based DGS simulation are shown in Figure 16. The implemented SCSRR demonstrated insignificant improvement in the isolation response compared to the coaxial probe feed MIMO antenna. Since each feeding network has a different current distribution on the ground plane that leads to the change in the impedance of the surface thus directly influencing the impedance matching.

**Figure 17:**
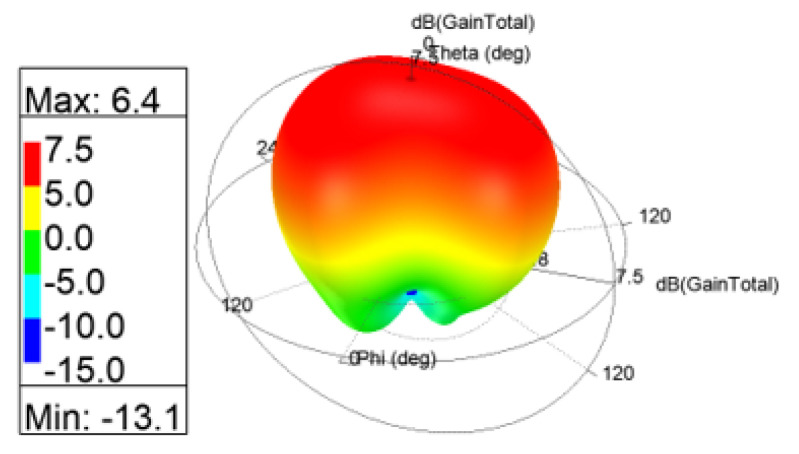
The 3D gain patterns of microstrip line feed MIMO antennas with SCSRR-based DGS.

**Figure 18:**
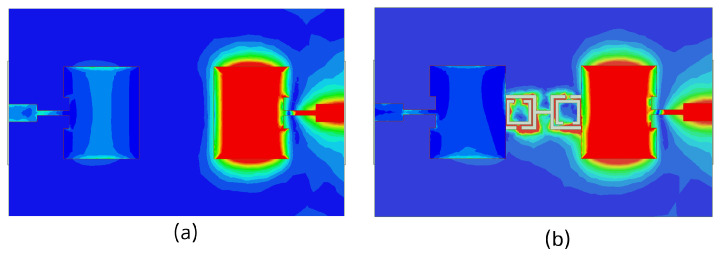
Current distribution of microstrip line feed MIMO antennas (**a**) without and (**b**) with SCSRR-based DGS.

The 3D gain patterns of the microstrip line feed MIMO antennas with SCSRR-based DGS is illustrated in Figure 17. The current distribution of microstrip line feed MIMO antennas with and without CSRR-based DGS at the 5.8 GHz resonance frequency is displayed in Figure 18. The surface current was not highly distributed on the microstrip line feed MIMO antenna with CSRR-based DGS (see Figure 18b) than without it because the capacitance and inductance effect had not been adequately increased. A comparison of microstrip line feed MIMO the antenna performance parameters with and without SCSRR-based DGS structures is provided in Table 5.

### 3.3. Proximity-Coupled Feed

**Figure 19:**
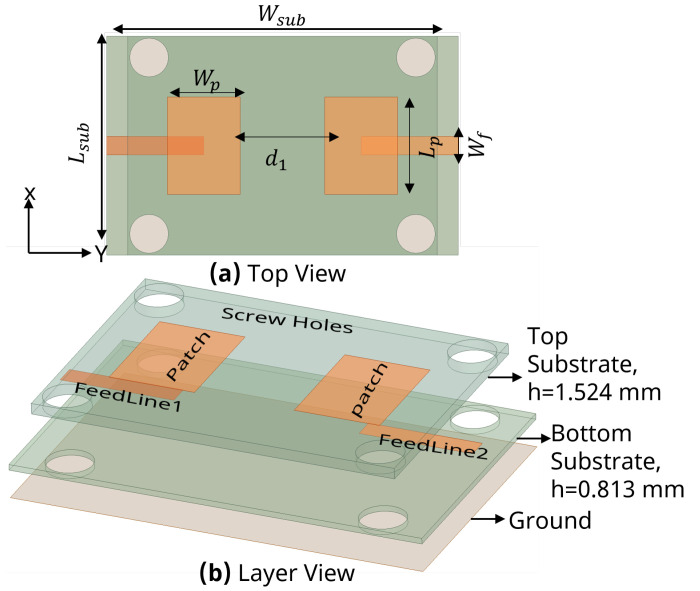
Proximity-coupled feed MIMO antenna model without SCSRR-based DGS. Dimensions of antenna: L_*sub*_ = 36 mm, W_*sub*_ = 58 mm, L_*p*_ = 16 mm, W_*p*_ = 13 mm, d_1_ = 26 mm, w_*f*_ = 3 mm, w_*t*_ = 0.8 mm.

**Figure 20:**
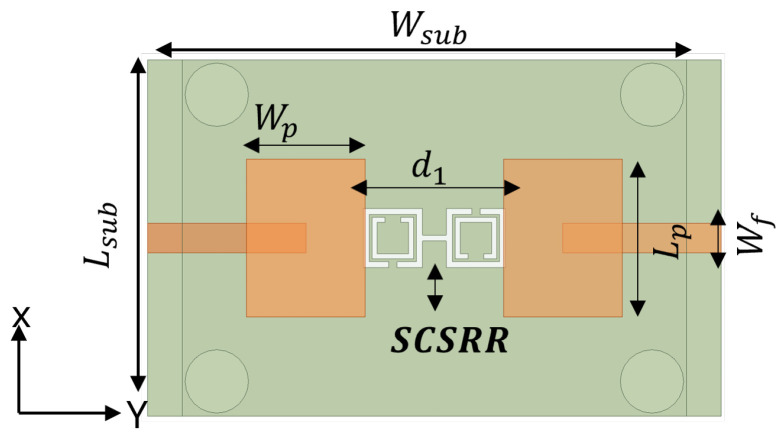
Proximity-coupled feed MIMO antenna model with SCSRR-based DGS. Note: Dimensions of SCSRR are listed in Table 3.

**Figure 21:**
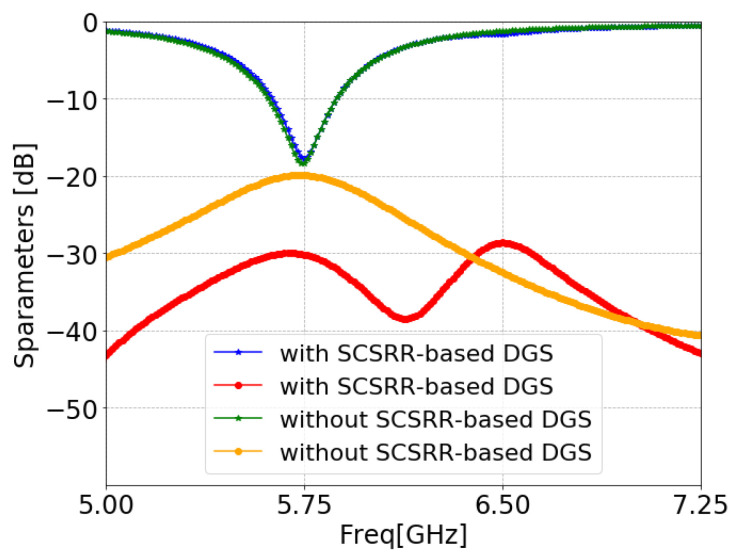
S-parameters of proximity-coupled feed MIMO antenna with and without SCSRR-based DGS.

The simulation model with dimensions of proximity-coupled feed MIMO antennas with and without SCSRR-based DGS structures are shown in Figure 19 and Figure 20, respectively. In order to obtain the impact of SCSRR-based DGS, MIMO antennas with and without SCSRR-based DGS have been analyzed, as illustrated Figure 21. Notable influences of the SCSRR on the antenna isolation characteristic (close to 10 dB improvement) were observed, as shown in Figure 21.

**Figure 22:**
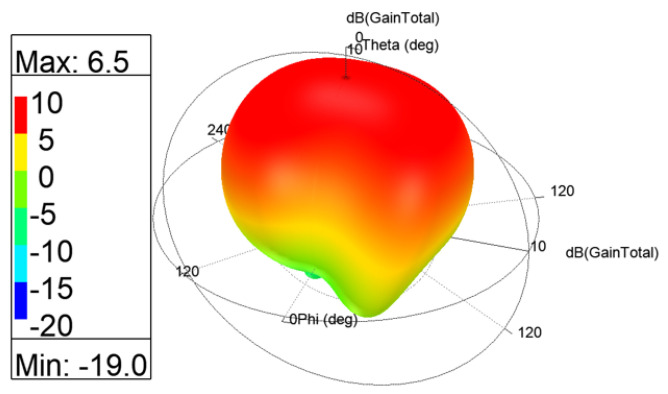
The 3D gain patterns of proximity-coupled feed MIMO antennas with SCSRR-based DGS.

**Figure 23:**
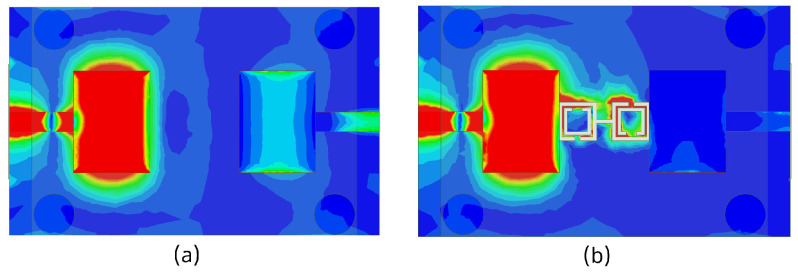
Current distribution of proximity coupled feed MIMO antennas (**a**) without and (**b**) with SCSRR-based DGS.

The 3D gain patterns of the proximity coupled feed MIMO antennas with SCSRR-based DGS are depicted in Figure 22. A decrease in the main lobe was noticed (see Figure 22) due to the presence of the SCSRR-based DGS. Figure 23 shows the surface current distribution for proximity feed MIMO antennas at 5.8 GHz. It is clearly observed from the distributions that the surface current of MIMO elements without SCSRR-based DGS has been suppressed after applying the SCSRR-based DGS structure. A comparison of the antenna performance parameters with and without SCSRR-based DGS structures is provided in Table 6.

### 3.4. Aperture-Coupled Feed

**Figure 24:**
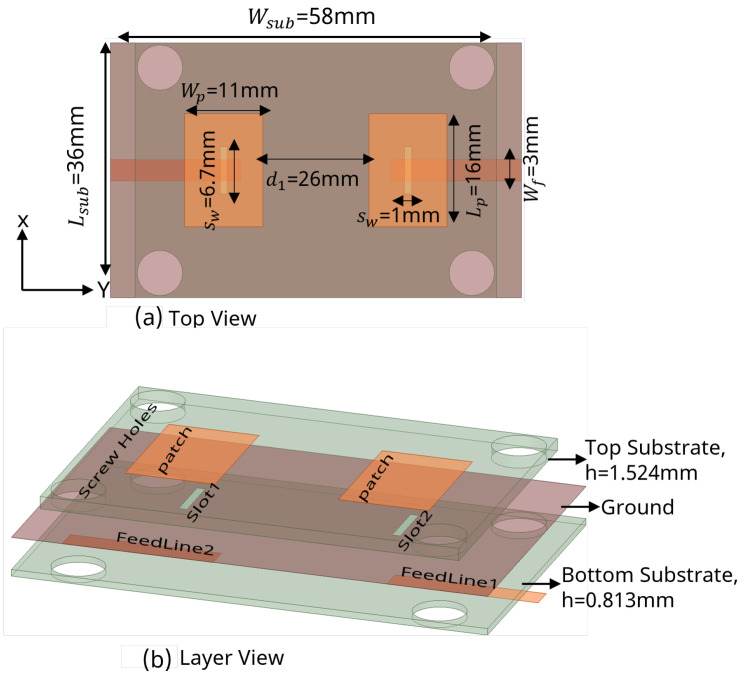
Dimensions of aperture-coupled feed MIMO antenna model without SCSRR-based DGS.

**Figure 25:**
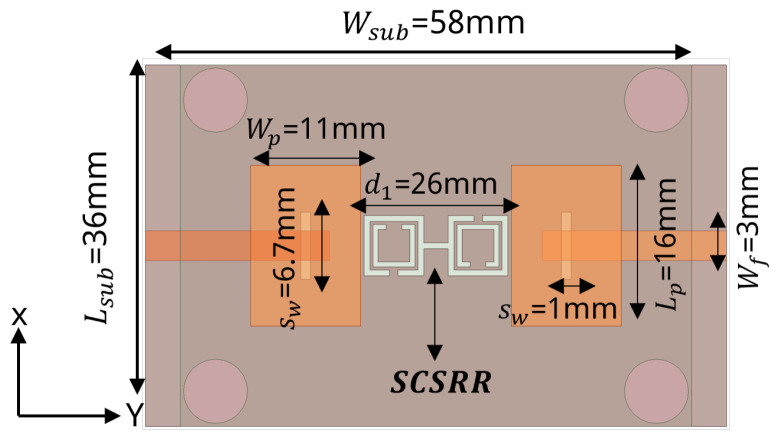
Dimensions of aperture coupled feed MIMO antenna model with SCSRR-based DGS.

**Figure 26:**
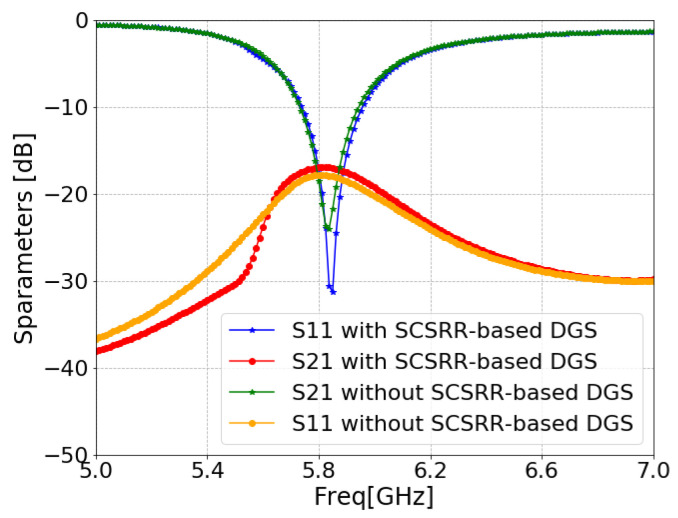
S-parameters of aperture coupled feed MIMO antennas with and without SCSRR-based DGS.

The schematics and dimensions of aperture-coupled feed MIMO antennas with and without SCSRR-based DGS are shown in Figure 24 and Figure 25, respectively. Figure 26 illustrates the simulated reflection coefficient ($S_{11}$) and transmission coefficient ($S_{21}$) characteristics of the aperture-coupled MIMO antennas with and without SCSRR-based DGS. From the result comparison, it can be noticed that the SCSRR-based DGS structure did not have significant impact on the antenna isolation for this specific feed mechanism.

**Figure 27:**
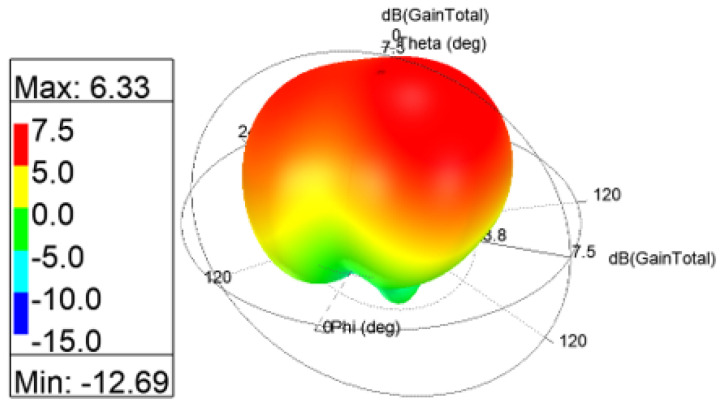
The 3D gain patterns of aperture-coupled feed MIMO antennas with SCSRR-based DGS.

**Figure 28:**
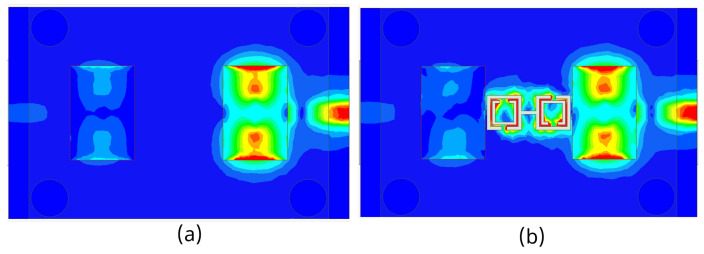
Current distribution of aperture-coupled feed MIMO antennas (**a**) without and (**b**) with SCSRR-based DGS.

To understand the radiation behavior of the aperture feed MIMO antennas, the 3D gain patterns were simulated, as shown in Figure 27. A slightly tilted pattern was yielded with the SCSRR-based DGS arrangement. The surface current distributions of aperture coupled feed MIMO antennas with and without SCSRR-based DGS are shown in Figure 28. Inadequate reduction in the concentration of coupling currents between MIMO antennas were noticed due to the SCSRR-based DGS. A comparative analysis of the MIMO antenna performance with and without SCSRR-based DGS is provided in Table 7.

### 3.5. MIMO Antenna Performance

**Figure 29:**
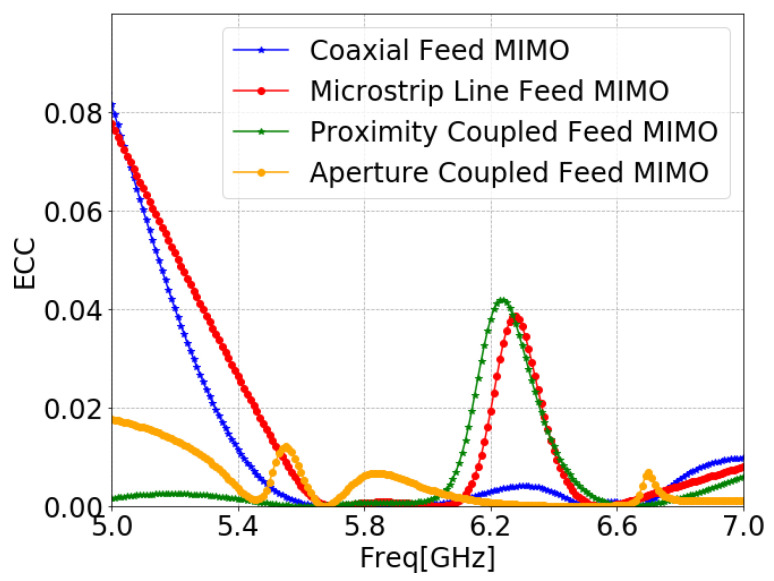
ECC of MIMO antennas with SCSRR-based DGS for each feeding methods.

The ECC is used to define how one antenna is correlated with other antennas present in their region of interference and it is calculated using Equation (7) [99,100]. ECC was below 0.05 in the operating frequency band, as shown in Figure 29, indicating that the proposed MIMO antenna is a good candidate for practical applications.

(7) $$ECC=\frac{|\int \int_{0}^{4\pi }\left[\vec{F_{i}}\left(\theta ,\phi \right)\times \vec{F_{j}}\left(\theta ,\phi \right)d\Omega \right]{|}^{2}}{\int \int_{0}^{4\pi }|\vec{F_{i}}\left(\theta ,\phi \right){|}^{2}d\Omega \int \int_{0}^{4\pi }|\vec{F_{j}}\left(\theta ,\phi \right){|}^{2}d\Omega }$$

where $\vec{F_{i}}\left(\theta ,\phi \right)$ and $\vec{F_{j}}\left(\theta ,\phi \right)$ indicates, respectively, the $i^{th}$ and $j^{th}$ elements of the antenna’s radiation patterns.

**Figure 30:**
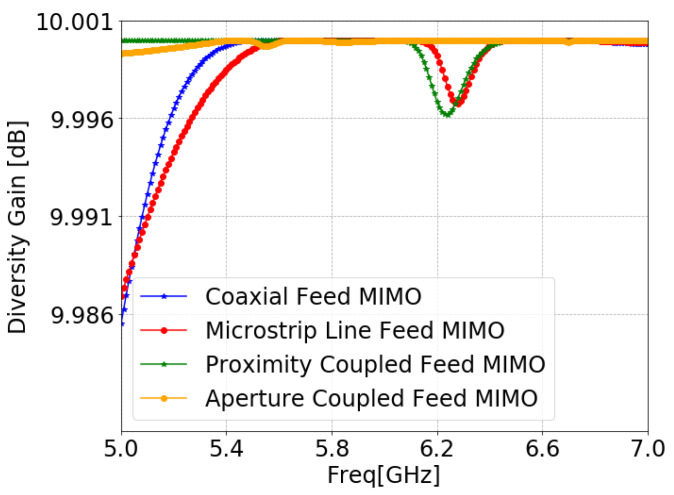
DG of MIMO antennas with SCSRR-based DGS for each feeding methods.

DG describes the amount of improvement of MIMO configuration that is estimated by using Equation (8) [100]. DG was close to 10 dB in the operating frequency band, as shown in Figure 30, confirming that the proposed MIMO antenna is an adequate option for real-world applications.

(8) $$DG=10\sqrt{1-{\left|\rho_{eij}\right|}^{2}}$$

**Figure 31:**
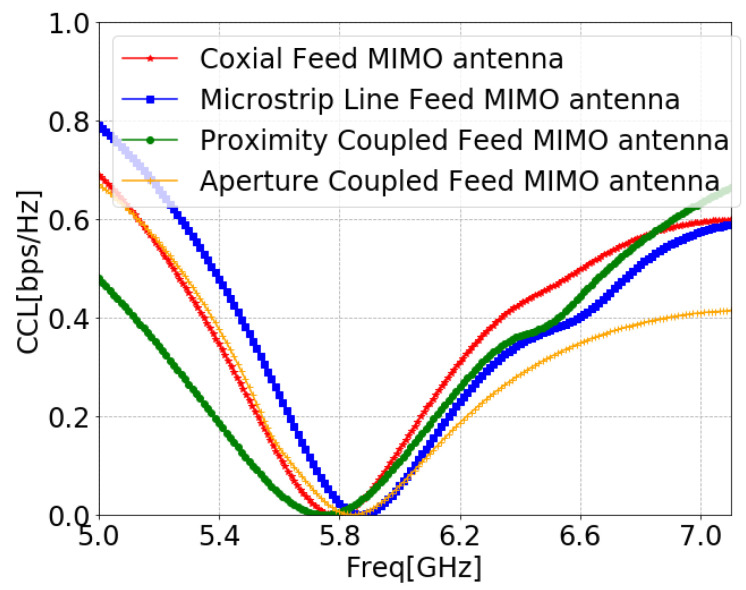
CCL of MIMO antennas with SCSRR-based DGS for each feeding methods.

The CCL details the channel capacity losses of the system during the correlation effect, and it is evaluated by Equations (9)–(12) [100]. Figure 31 proves that CCL was within the limits of practical standard of 0.4 bit/s/Hz [101] for the entire operating band, which justifies the proposed system’s successful transmission at the operating frequency.

(9) $$CCL=log_{2}det\left(\alpha \right),$$

(10) $$\alpha =\left[\begin{array}{cc} \sigma_{ii} & \sigma_{ij}\\ \sigma_{ji} & \sigma_{jj} \end{array}\right]$$

(11) $$\begin{array}{c} \sigma_{ii}=1-\left({\left|S_{ii}\right|}^{2}-{\left|S_{ij}\right|}^{2}\right) \end{array}$$

(12) $$\begin{array}{c} \sigma_{ij}=\left(S_{ii}^{*}S_{ij}+S_{ji}S_{jj}^{*}\right), \end{array}$$

**Figure 32:**
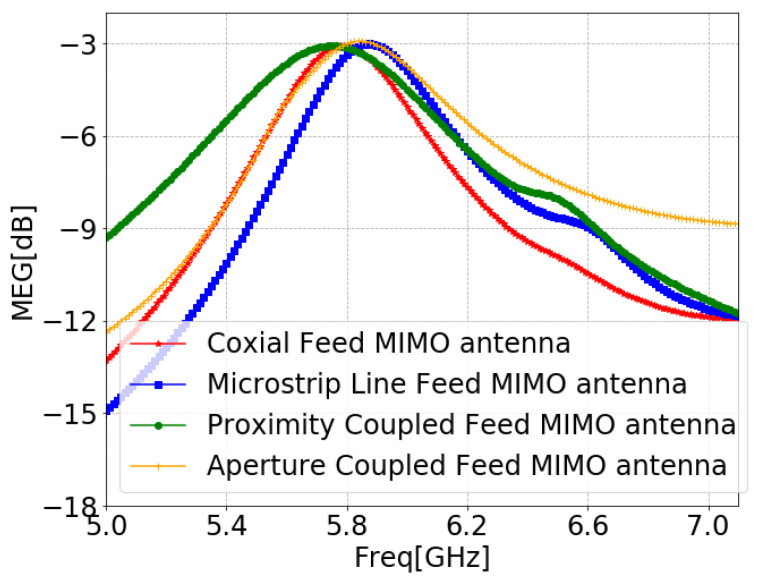
MEG of MIMO antennas with SCSRR-based DGS for each feeding methods.

The MEG is defined as the mean received power in the fading environment, and it is calculated using Equation (13) [100]. For acceptable diversity performance of the MIMO, the MEG needs to be between −3 dB and −12 dB [101], which is validated for the obtained MEG values of all MIMO antennas of the proposed designs, as demonstrated in Figure 32.

(13) $$MEG=0.5(1-\sum_{j=1}^{N}\left|S_{ij}\right|)$$

## 4. Experimental Validation

**Figure 33:**
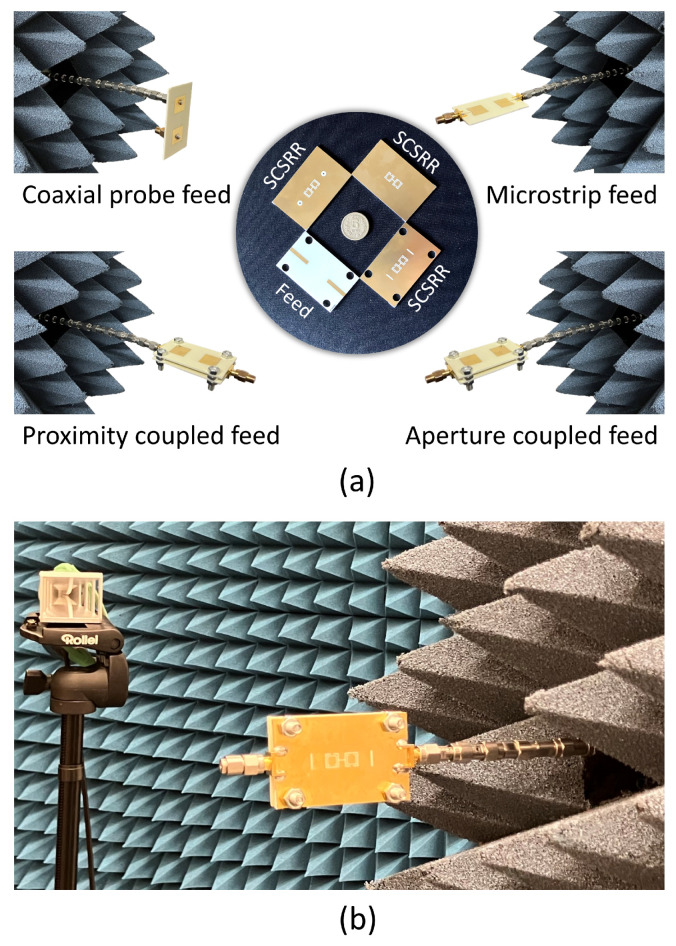
Photographs of the fabricated MIMO antennas with SCSRR-based DGS for (**a**) different feed networks and (**b**) aperture-coupled feed.

Four MIMO antennas based on the coaxial probe, microstrip line, proximity coupled, and aperture coupled feed methods have been manufactured using the conventional photo-lithography technique in order to verify the simulation results. Photographs of the fabricated MIMO antenna designs are shown in Figure 33a. The anechoic chamber at Barkhausen Institut in Germany was used for the fabricated MIMO antenna measurements. Their transmission and reflection characteristics were measured using a Keysight N5224B PNA microwave network analyzer. A standard horn antenna was used to measure the gain patterns. While measuring the radiation parameters of the MIMO antennas, a 50 Ω termination resistance was used. Two different antenna inclinations were used for radiation pattern measurements: vertical and horizontal in the port direction. A photograph of the proposed antennas under measurement in the anechoic chamber is demonstrated in Figure 33b. A substantial portion of the sets of observations showed good agreement between the measured and simulated results for the fabricated MIMO antennas. Similar effects were observed when one port of the fabricated MIMO antenna was stimulated whereas the remaining port was 50 Ω terminated. However, simulation to measurement differences have been noticed in the isolation results. The primary causes of the discrepancy include undesirable electromagnetic radiation through the feed cable, incorrect alignment, connector or 50 Ω termination resistance tolerances, and errors in the production phase.

**Figure 34:**
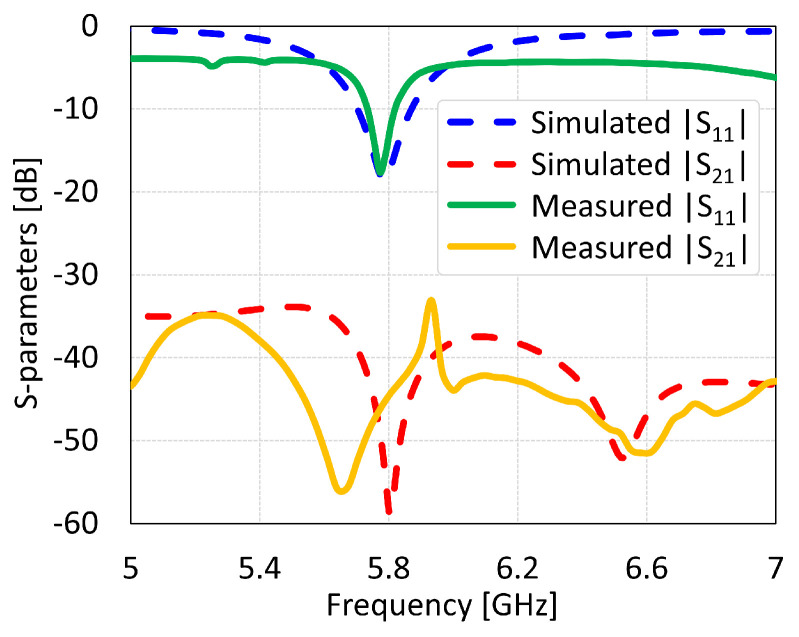
Simulated and measured S-parameters of coaxial feed MIMO antenna with SCSRR-based DGS.

**Figure 35:**
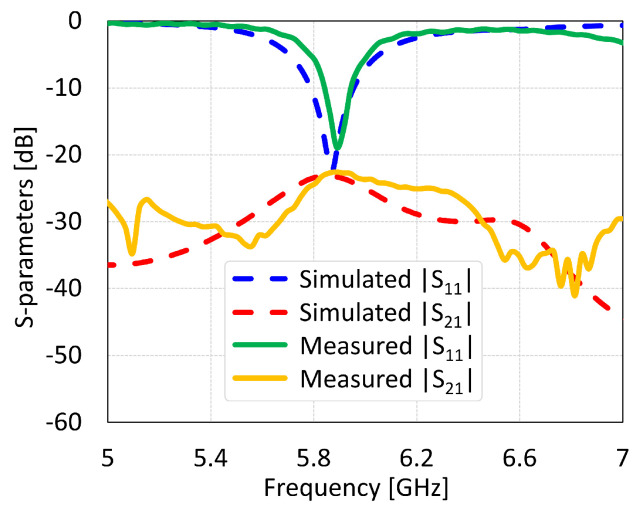
Simulated and measured S-parameters of microstrip line feed MIMO antenna with SCSRR-based DGS.

**Figure 36:**
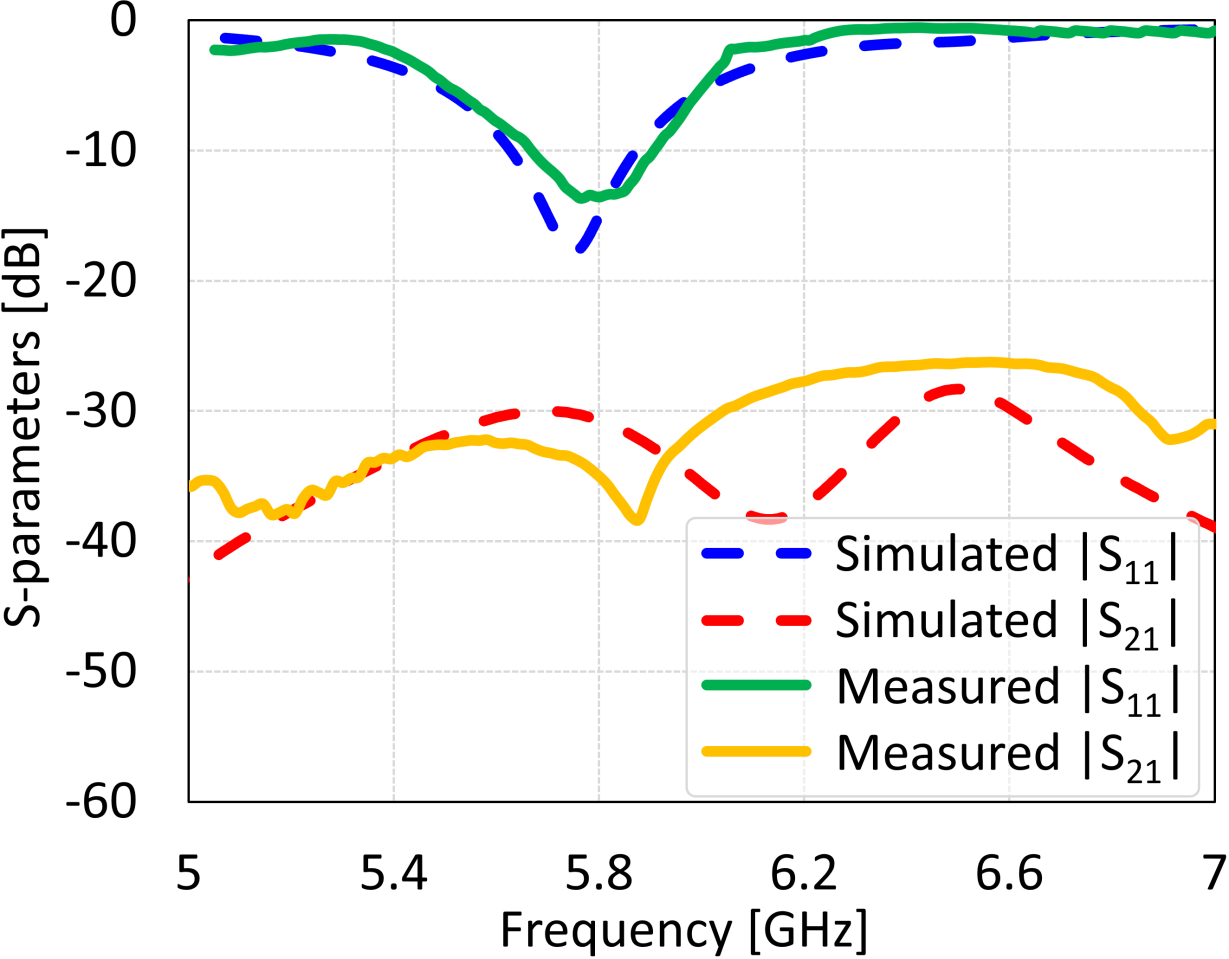
Simulated and measured S-parameters of proximity coupled feed MIMO antenna with SCSRR-based DGS.

**Figure 37:**
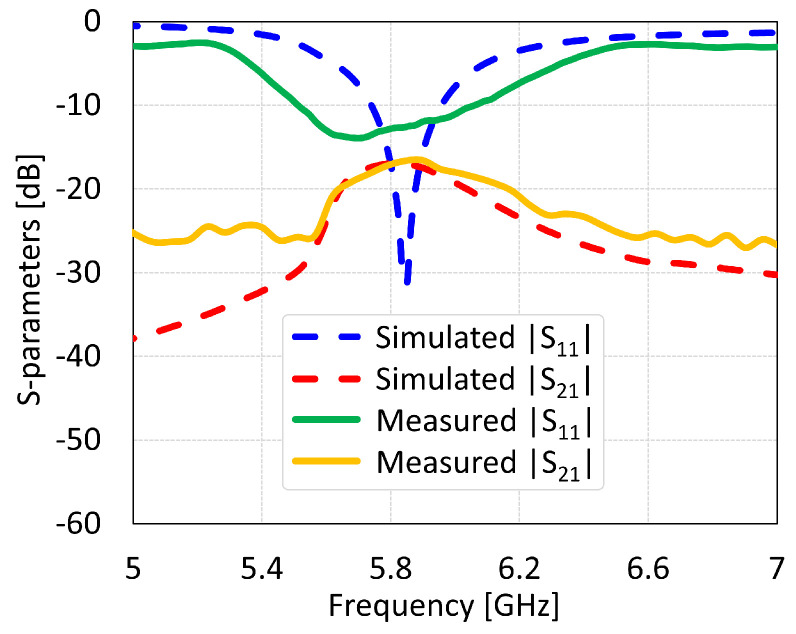
Simulated and measured S-parameters of aperture coupled feed MIMO antenna with SCSRR-based DGS.

The MIMO antennas showed measured BW and isolation values of 150 MHz and 46 dB for coaxial feed, 125 MHz and 22 dB for microstrip line feed, 312 MHz and 38 dB for proximity coupled feed, and 312 MHz and 17 dB for aperture coupled feed, as shown in Figure 34, Figure 35, Figure 36 and Figure 37, respectively. The discrepancies in the simulated and measured isolation are attributable to the electromagnetic radiations from the feed cable. Note that the measured peak isolation values is concerned only in −10 dB matching area and then too, the number for coaxial probe feed antenna was relatively better. As it is seen from the comparison, there is are different frequency shifts between S_1_1 parameters for measurement and simulation. That could occur because of the calibration of equipment, soldering of feed mechanism (which directly affects impedance matching) as well as different surface roughness of the antennas.

**Figure 38:**
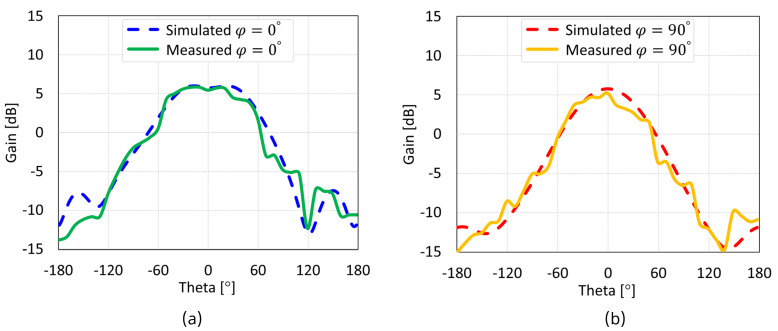
Simulated and measured radiation patterns of coaxial feed MIMO antenna with SCSRR-based DGS: (**a**) E-plane; (**b**) H-plane.

**Figure 39:**
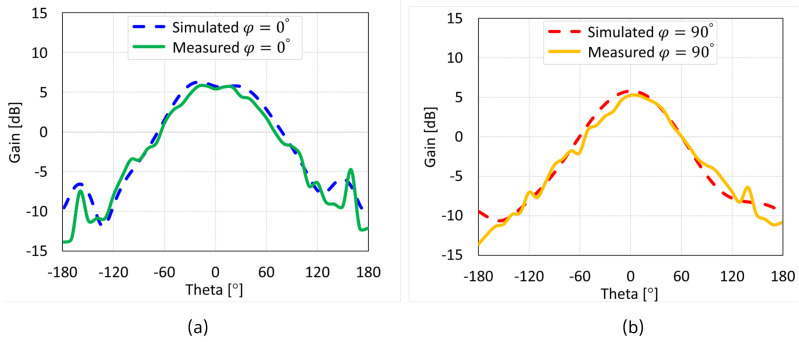
Simulated and measured radiation patterns of microstrip line feed MIMO antenna with SCSRR-based DGS: (**a**) E-plane; (**b**) H-plane.

**Figure 40:**
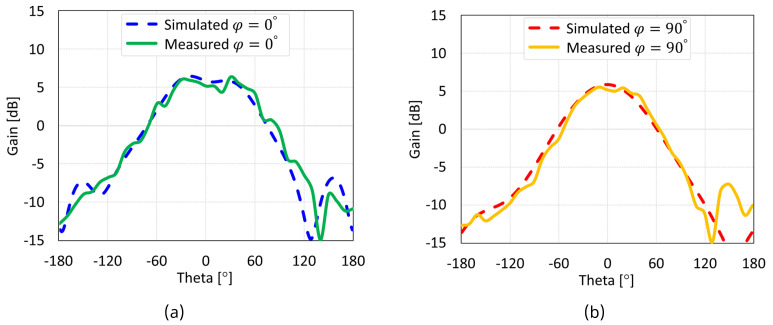
Simulated and measured radiation patterns of proximity coupled feed MIMO antenna with SCSRR-based DGS: (**a**) E-plane; (**b**) H-plane.

**Figure 41:**
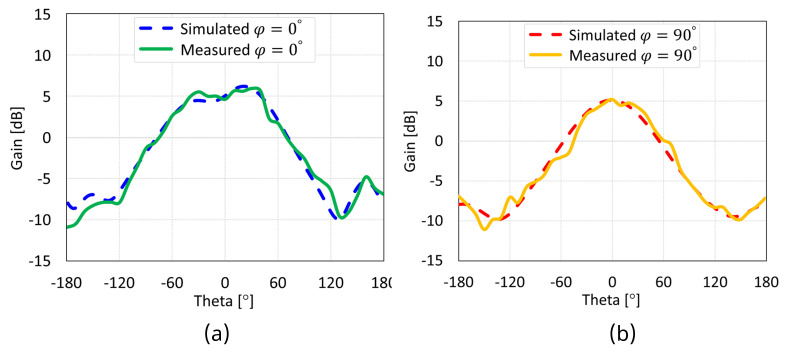
Simulated and measured radiation patterns of aperture-coupled feed MIMO antenna with SCSRR-based DGS: (**a**) E-plane; (**b**) H-plane.

The simulated and measured radiation patterns of the MIMO antennas with coaxial probe, microstrip line, proximity-coupled, and aperture-coupled feed methods at 5.8 GHz are displayed in Figure 38, Figure 39, Figure 40, and Figure 41, respectively. Nearly omnidirectional patterns was noticed. In the operational frequency band of every MIMO antenna, the realized gain was ≈5 dBi.

## 5. Benchmarking

The effects of four popular feed networks—coaxial probe, microstrip line, proximity-coupled, and aperture-coupled feed—on a traditional patch antenna with a full ground plane were thoroughly investigated in [102]. The results of this research indicated that the proximity coupled feed approach stands out in achieving a wide bandwidth, and all feeding methods’ gain values showed some variation, with the highest gain deviation value being ≈1.7 dBi. However, our results in Table 8 indicate that all feeding techniques have a relatively different effect on the behavior of MIMO antennas integrated with SCSRR-based DGS. A variation of behavior arises from the integrated DGS and distinct feeding network. As it is widely known, an integrated DGS structure significantly affects the impedance-matching circuit in several ways such as modifying the effective permittivity and permeability of the substrate. Depending on the geometry, the DGS structure acts as an inductive and capacitive, resulting in input impedance changes in the antenna. The proposed SCSRR-based DGS induces electromagnetic force that generates a current flow within the metallic rings and gaps, producing a balanced inductive–capacitive effect. Accordingly, the selection of a feeding technique for the MIMO antenna is also crucial since it affects key performance parameters like radiation efficiency, BW, and impedance matching. Therefore, to design a high-performance MIMO antenna integrated with DGS, two essential criteria come to the forefront, such as choosing a proper feeding network and a suitable DGS-shaped device, which have direct impacts on the impedance matching circuit.

Particularly, both proximity-coupled and aperture-coupled feed methods are advantageous for wideband applications, and all feeding methods offer similar gain values. Furthermore, our research adds light on the isolation values. In [103], a microstrip line fed MIMO antenna with SCSRR-based DGS was proposed for isolation enhancement. However, the design was limited to achieving a maximum isolation of 27 dB. Similarly, several other MIMO antenna designs for isolation enhancement have focused on SCSRR-inspired DGS with a microstrip line feed, as evident in [104,105,106]. Our research demonstrates that a coaxial probe feed technique is capable of attaining relatively high peak isolation (48 dB see in Figure 38) without complicating the MIMO antenna design due to their feed network and DGS-shape being compatible and providing good impedance matching. Table 9 presents a comparison of simulation results between the feed networks of the reference antennas and our recommended configurations. Our simulations and measurements confirm that integrating a precisely designed DGS, coupled with optimized feed network configurations, enhances key performance parameters of the MIMO antenna, as demonstrated in the proposed coaxial probe-fed MIMO antenna. It may be concluded that the choice of antenna feed type has received less attention over the years. Thus, our study provides valuable insights to assist future researchers in choosing the appropriate feed type according to their intended applications, and it constitutes the work’s original contribution.

## 6. Conclusions

This paper explores MIMO antennas integrated with SCSRR-based DGS across various feed networks, comparing their performance. Four standard feed methods were carefully evaluated in terms of MIMO antenna performance parameters. The primary goal was to identify and address a research gap: the lack of performance analysis for different feed networks using identical DGS structures, offering valuable insights for future investigators. Initially, it was thought that antennas with DGSs would operate similar to those without DGSs. Nevertheless, a detailed study revealed that the DGS structure significantly impacts the antenna bandwidth and isolation, with proximity- or aperture-coupled feed methods proving more suitable for wideband applications. Furthermore, coaxial probe and microstrip line feed approaches excel in high isolation and straightforward fabrication. Thus, optimizing the DGS shape and dimensions is crucial to match specific feeding technique requirements. Moreover, it is noted that researchers often rely on the time-intensive trial-and-error method to examine ground plane current distribution for DGS benefits. Alternatively, evaluating the dispersion diagram of a unit DGS cell offers a quicker approach to determine the EBG range. Future research avenues could involve developing standard equations to predict DGS architectures, benefiting various disciplines. Given DGS’s cost-effectiveness in enhancing MIMO antenna performance, its application extends to the design of other radio frequency circuits. 

## Figures and Tables

**Table 1:** Advantages and disadvantages of different antenna feed networks.

Feed Methods	Advantages	Disadvantages
Microstrip Line	Easy fabrication and modeling. Easy integration with other RF components on the same substrate. Simplicity for modeling impedance matching and low-profile design.	Limited bandwidth. Spurious feed radiation may occur from surface wave. Transmission line may cause interference in designs.
Coaxial Probe	Easy fabrication. Suitable for high permittivity and thick substrates. Direct connection to external circuity (e.g., transceivers). Low spurious feed radiation. Higher antenna RE.	Limited bandwidth. Difficult modeling. Integration complexity due to the need for vertical integration.
Aperture Coupled	Easy modeling. Enhancement of gain and BW. Low spurious feed radiation.	Difficulty in modeling impedance matching. Complex fabrication. Backward radiation due to aperture.
Proximity-Coupled	Easy modeling. Enhancement of gain and BW. Low spurious feed radiation.	Difficulty in modeling impedance matching. Complex fabrication.

**Table 2:** Advantages and disadvantages of defected ground structure-based antennas [29,91,92,93,94,95,96].

Advantages	Disadvantages
Suppression of surface wave	Backward radiation
Harmonic suppression	Difficulty in analysis
Enhancement of bandwidth	Difficulty in design
Easy fabrication and cost effective	Radiation pattern distortion

**Table 3:** Dimensions of the SCSRR.

Parameters	Values [mm]
L_*out*_	6
g_*out*_	0.3
t_*out*_	0.6
d_*in*_	1.6
S_*L*_	2.4
S_*W*_	0.6
L_*in*_	4.2
t_*in*_	0.5
d_*out*_	0.85

**Table 4:** Performance comparison of coaxial probe feed MIMO antennas with and without SCSRR-based DGS.

Parameters	Without SCSRR	With SCSRR
$f_{c}$ [GHz]	5.76	5.8
Peak return loss [dB]	19.15	17.67
BW [MHz]	170	180
Peak gain [dBi]	6.8	6
Peak isolation [dB]	16.5	58
RE [%]	95.9	95.8

**Table 5:** Performance comparison of microstrip line feed MIMO antennas with and without SCSRR-based DGS.

Parameters	Without SCSRR	With SCSRR
$f_{c}$ [GHz]	5.85	5.87
Peak return loss [dB]	19	21.86
BW [MHz]	162	175
Peak gain [dBi]	7.1	6.4
Peak isolation [dB]	21	24
RE [%]	94.4	94.3

**Table 6:** Performance comparison of proximity coupled feed MIMO antenna with and without SCSRR-based DGS.

Parameters	Without SCSRR	With SCSRR
$f_{c}$ [GHz]	5.75	5.75
Peak return loss [dB]	18	17.78
BW [MHz]	263	270
Peak gain [dBi]	7.2	6
Peak isolation [dB]	20	31
RE [%]	97.4	97.2

**Table 7:** Performance comparison of aperture-coupled feed MIMO antennas with and without SCSRR-based DGS.

Parameters	Without SCSRR	With SCSRR
$f_{c}$ [GHz]	5.84	5.85
Peak return loss [dB]	24	31
BW [MHz]	210	225
Peak gain [dBi]	6.3	6.33
Peak isolation [dB]	18	17
RE [%]	94.9	94.8

**Table 8:** Impact of different feed networks on the performance of MIMO antennas with SCSRR DGS.

Parameters	Coaxial Probe		Microstrip Line		Proximity Coupled		Aperture Coupled	
Experiment	Simulated	Measured	Simulated	Measured	Simulated	Measured	Simulated	Measured
$f_{c}$ [GHz]	5.8	5.8	5.87	5.87	5.75	5.75	5.85	5.85
BW [MHz]	180	150	175	125	270	312	225	312
Peak gain [dBi]	6	≈5	6.4	≈5	6	≈5	6.33	≈5
Peak isolation [dB]	58	46	24	22	31	38	17	17

**Table 9:** Feed mechanism employed by state-of-the-art DGS-based antennas.

Reference	Aim: To Enhance	Accomplishment	Utilized Feed Type	Recommende Feed Type: by Us
[107]	Isolation	25 dB	Microstrip line	Coaxial probe
[108]	Isolation	15 dB	Microstrip line	Coaxial probe
[109]	Isolation	18.7 dB	Microstrip line	Coaxial probe
[110]	Isolation	18 dB	Microstrip line	Coaxial probe
[111]	Bandwidth	260 MHz	Microstrip line	Aperture or proximity coupled

## Data Availability

The data supporting the reported results in this study are not available due to confidentiality restrictions.
